# Necrology

**Published:** 1897-05

**Authors:** 


### NECROLOGY.

Lucian T. Bell, M.D., V.S., died on Friday morning, April 16th, the result of failing health, which had been making inroads on his physical condition for the past year.

Dr. Bell was born at Staunton, Va., May 18, 1850, and received his early education in the country academical schools; he graduated from the old New York College of Veterinary Surgeons in 1871, the class of that year consisting of but four students, only two of whom were successful in receiving a diploma. After the establishment of the American Veterinary College he returned, and with some others received the degree of that school in 1876, and in 1880 finished a course in human medicine and received the diploma of the Long Island Medical College. He served under General Patrick in the first crusade against pleuro-pneumonia in 1876, as chief inspector for Long Island. When the Bureau of Animal Industry inaugurated the movement to suppress pleuro-pneumonia he was again summoned to aid in this work, and served until the Harrison administration. From 1885 to 1894 he was veterinarian to the Brooklyn Board of Health. In 1875 he became a member of the United States Veterinary Medical Association, but relinquished his membership some years later, owing to a very large practice and the care of a model veterinary sanitarium at 358 South Second Street, Brooklyn, E. D. Dr. Bell was a man of spotless integrity, an appreciated practitioner by a very large clientage, and one of the most successful in the city of Brooklyn. A wife and four children are left to mourn his loss, which will be mingled with the sympathy and sorrow of everyone who was fortunate to have enjoyed his friendship and acquaintance.

---

It is very encouraging to the efforts made by the editorial staff of the Journal to receive the following in a letter sent to the editorial office, and we assure the writer of our sincere appreciation: "The March number of the Journal came promptly to hand, and is replete with articles interesting to all veterinarians. The industry, energy, and untiring perseverance employed in the production of this Journal are little appreciated, I fear, by the casual reader; but everyone who has noted the growth of this and other veterinary periodicals ought to take in it a personal pride and use his best endeavors to increase its subscription-list and its scope of usefulness. I am appalled with amazement when I think of the amount of work given by somebody to furnish me with such a valuable periodical. May the subscriptions justify the outlay of energy."

